# Point-of-care testing in out-of-hospital cardiac arrest: a retrospective analysis of relevance and consequences

**DOI:** 10.1186/s13049-021-00943-w

**Published:** 2021-08-30

**Authors:** Tobias Gruebl, B. Ploeger, E. Wranze-Bielefeld, M. Mueller, W. Schmidbauer, C. Kill, S. Betz

**Affiliations:** 1 Department of Anaesthesiology, Intensive Care, Emergency Medicine and Pain Therapy, Bundeswehr Central Hospital, Ruebenacher Straße 170, 56072 Koblenz, Germany; 2 Center of Emergency Medicine, University Hospital of Marburg, Baldingerstraße, 35043 Marburg, Germany; 3 Department of Hazard Prevention and Emergency Service, District of Vogelsberg, Goldhelg 20, 36341 Lauterbach, Germany; 4 German Red Cross Emergency Service of Mittelhessen gGmbH, Am Krekel 41, 35039 Marburg, Germany; 5 Center of Emergency Medicine, University Hospital of Essen, Hufelandstraße 55, 45147 Essen, Germany

**Keywords:** Point-of-care-testing, POCT, Out-of-hospital cardiac arrest, OHCA, Resuscitation, H’s and T’s, Hyperkalaemia, Hypokalaemia, Blood gas analysis

## Abstract

**Background:**

Metabolic and electrolyte imbalances are some of the reversible causes of cardiac arrest and can be diagnosed even in the pre-hospital setting with a mobile analyser for point-of-care testing (POCT).

**Methods:**

We conducted a retrospective observational study, which included analysing all pre-hospital resuscitations in the study region between October 2015 and December 2016. A mobile POCT analyser (Alere epoc®) was available at the scene of each resuscitation. We analysed the frequency of use of POCT, the incidence of pathological findings, the specific interventions based on POCT as well as every patient’s eventual outcome.

**Results:**

N = 263 pre-hospital resuscitations were included and in n = 98 of them, the POCT analyser was used. Of these measurements, 64% were performed using venous blood and 36% using arterial blood. The results of POCT showed that 63% of tested patients had severe metabolic acidosis (pH < 7.2 + BE <  − 5 mmol/l). Of these patients, 82% received buffering treatment with sodium bicarbonate. Potassium levels were markedly divergent normal (> 6.0 mmol/l/ < 2.5 mmol/l) in 17% of tested patients and 14% of them received a potassium infusion. On average, the pre-hospital treatment time between arrival of the first emergency medical responders and the beginning of transport was 54 (± 20) min without POCT and 60 (± 17) min with POCT (*p* = 0.07). Overall, 21% of patients survived to hospital discharge (POCT 30% vs no POCT 16%, *p* = 0.01, Φ = 0.16).

**Conclusions:**

Using a POCT analyser in pre-hospital resuscitation allows rapid detection of pathological acid–base imbalances and potassium concentrations and often leads to specific interventions on scene and could improve the probability of survival.

## Background

Sudden cardiac arrest remains one of the main causes of death in Western industrialised countries [1, 2]. Even with the best emergency medical care, only about 40% of patients who experience out-of-hospital cardiac arrest (OHCA) survive with return of spontaneous circulation (ROSC) to hospital admission and the average rate of survival to discharge in the Western world is less than 10% [3–8].

The relevant guidelines highlight the importance of early detection and treatment of potentially reversible causes of cardiac arrest and explicitly mention potassium imbalances and other metabolic imbalances in this regard. They recommend point-of-care testing (POCT) so that specific therapeutic measures can be taken if pathological values are found [9, 10].

Portable POCT devices are increasingly available today and so parameters such as electrolyte concentrations, lactate and blood glucose can quickly be determined at the site of the emergency in addition to the parameters of blood gas analysis such as pH value, partial gas pressures, base excess and bicarbonate concentration [11, 12]. Such testing thus facilitates early, targeted treatment even before arrival at the hospital.

There are few studies available on changes in blood parameters determined in OHCA patients in the pre-hospital setting. We examined the effects of the on-scene availability of mobile POCT equipment in a physician-staffed emergency medical service.

## Methods

### Research methods

Once we had obtained the approval of the ethics committee (ref. no.: 86/16, University of Marburg), we retrospectively evaluated the POCT results of OHCA patients treated between October 2015 and December 2016. The standard emergency response for resuscitation cases in Germany and thus also in the study region (1263 km^2^, 199 inhabitants per km^2^) is to send out an emergency medical service unit with three paramedics and an emergency physician. Unusually at the time, every unit in the study region carried a POCT analyser (Alere epoc®, results available within approx. 3 min). All of these vehicles were operated by the same organisation and all staff had received the same training in advanced life support in accordance with the current applicable guidelines. There is only one hospital with a cardiac arrest centre in the study region, so, with very few exceptions, the resuscitation patients in our study were taken there. We collected the data for our evaluation from the ambulance reports and directly from the measuring instruments. It was anonymised before being stored and analysed.

We only used data from patients aged 18 years or older. Because of the retrospective design of the study, there were no study-specific instructions on the use of POCT in OHCA.

### Statistics

Depending on the scale level and the form of variable distribution, the mean value or median (central tendency), minima and maxima (extreme values) as well as standard deviation and interquartile ranges (measures of dispersion) were calculated for descriptive statistical analysis.

Frequency differences were tested for significance using the 2 × 2 contingency chi-squared test and, in the case of low cell occupancy, the Fisher’s exact test.

In the individual patient groups with and without POCT analysis, differences in central tendency were assessed for significance using the *t*-test for independent samples and using the Mann–Whitney *U*-test for non-parametric differences.

### Endpoints

The primary endpoint was defined as the frequency and severity of metabolic and electrolyte imbalances in the point-of-care test results. The secondary endpoints were specific interventions by emergency physicians, time taken for POCT results to be available in practice, pre-hospital treatment time as well as course and outcome of further treatment. The Pre-Emergency Status (PES) was used to classify the physical status of every patient prior to their OHCA (1 = normal healthy patient, 2 = patient with mild systemic disease, 3 = patient with severe systemic disease and substantive functional limitations, 4 = patient with severe systemic disease that is a constant threat to life, 5 = patient who is not expected to survive the next 24 h, regardless of medical treatment provided).

## Results

A total of n = 263 pre-hospital resuscitations were included. In n = 98 (37%) of these cases, the emergency physician on the scene used a POCT analyser. Detailed baseline patient data, POCT measurements, treatment times, and outcome are included in Table 1. In n = 16 cases where arterial blood samples were taken, the puncture site was also documented (94% radial artery, 6% femoral artery).

**Table 1:** Data of the OHCA patients

	Total		POCT		No POCT		
*Source data*							
n	263		98 (37%) Arterial 35 (36%)/venous 63 (64%)		165 (63%)		
Mean age (years)	70 (± 13)		69 (± 14)		70 (± 13)		*p* = 0.86
Gender	185 male (70%)	78 female (30%)	73 male (74%)	25 female (26%)	112 male (68%)	53 female (32%)	*p* = 0.26
Mean number patients with known pre-existing diseases	1.3 (± 0.9)		1.2 (± 0.9)		1.3 (± 1.0)		*p* = 0.15
Mean PES	2.57 (± 0.8)		2.5 (± 0.8)		2.6 (± 0.7)		*p* = 0.13
Observed collapse	149 (57%)		61 (62%)		88 (53%)		*p* = 0.20
Initial VF/pVT	69 (26%)		38 (39%)		31 (19%)		*p* < 0.001
Initial Asys/PEA	194 (74%)		60 (61%)		134 (81%)		*p* < 0.001
Cardiac cause suspected	181 (69%)		72 (73%)		109 (66%)		*p* = 0.21
*POCT*							
pH 7.35–7.45			4 (4%)				
pH < 7.35			91 (93%)				
pH < 7.35 + pCO_2_ > 50 [BE in norm]			2 (2%)				
pH < 7.35 + BE < − 2 [pCO_2_ in norm]			13 (13%)				
pH < 7.35 + pCO_2_ > 50 + BE < − 2			68 (69%)				
pH < 7.2			77 (79%)				
pH < 7.2 + BE < − 5 [regardless of CO_2_]			62 (63%)				
K^+^ > 5.2/K^+^ < 3.5 mmol/l			33 (34%)				
K^+^ > 5.2 mmol/l			22 (23%)				
K^+^ < 3.5 mmol/l			11 (11%)				
K^+^ > 6.0/K^+^ < 2.5 mmol/l			17 (17%)				
K^+^ > 6.0 mmol/l			16 (16%)				
K^+^ < 2.5 mmol/l			1 (1%)				
Lactate > 2.5 mmol/l			90 (92%)				
Glucose (1 mg/dl = 0.056 mmol/l)			> 200 mg/dl: 58 (59%)				
			< 50 mg/dl: 2 (2%)				
*Specific therapy*							
Bicarb 8.4%	84 (32%)		61 (62%)		23 (14%)		*p* < 0.001
Bicarb 8.4% in cases where, pH < 7.35 + BE < − 2			11 (18%)				
Bicarb 8.4% in cases where, pH < 7.2 + BE < − 5			50 (82%)				
K^+^-specific therapy	22 (8%)		22 (22%)		0 (0%)		
Calcium 10%			8 (36%)				
KCl 7.45%			14 (64%)				
KCl 7.45% + Bicarb 8.4%			10 (71%)				
*Treatment time*							
Mean treatment time (min)	57 (± 19)		60 (± 17)		54 (± 20)		*p* = 0.07
Mean time to POCT (min)			37 (± 21)				
POCT before ROSC			26 (± 15)				
*Outcome*							
ROSC	141 (54%)		72 (73%)		69 (42%)		*p* < 0.001
Hospital admission	163 (62%)		79 (81%)		84 (51%)		*p* < 0.001
With ROSC	122 (46%)		65 (82%)		57 (68%)		*p* < 0.001
Ongoing CPR	41 (16%)		14 (18%)		27 (32%)		*p* = 0.78
Discharged	55 (21%)		29 (30%)		26 (16%)		*p* = 0.01

PES, pre-emergency status; VF, ventricular fibrillation; VT, ventricular tachycardia; Asys, asystole; PEA, pulseless electrical activity; pH, potentia hydrogenii; BE, base excess; K^+^, potassium; Bicarb, sodium hydrogen carbonate; KCl, potassium chloride; POCT, point-of-care testing;ROSC, return of spontaneous circulation

Of the patients who underwent POCT, n = 62 (63%) were found to have severe acidosis with metabolic disturbance as a contributory cause (pH < 7.2 + BE <  − 5 mmol/l). There was no significant difference between patients with observed and unobserved collapse when it came to severe acidosis (observed + severe metabolic acidosis n = 34/56% vs unobserved + severe metabolic acidosis n = 28/76%, *p* = 0.054).

**Fig. 1:**
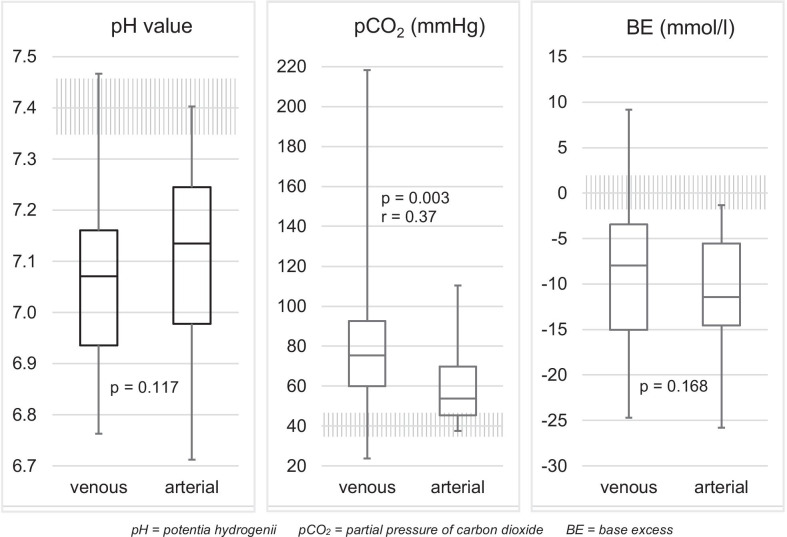
Acid–base status in POCT results of OHCA patients (upper/lower whiskers: maximum/minimum value; box: 75% to 25% quartile; median as line in the box. Normal ranges hatched)

**Fig. 2:**
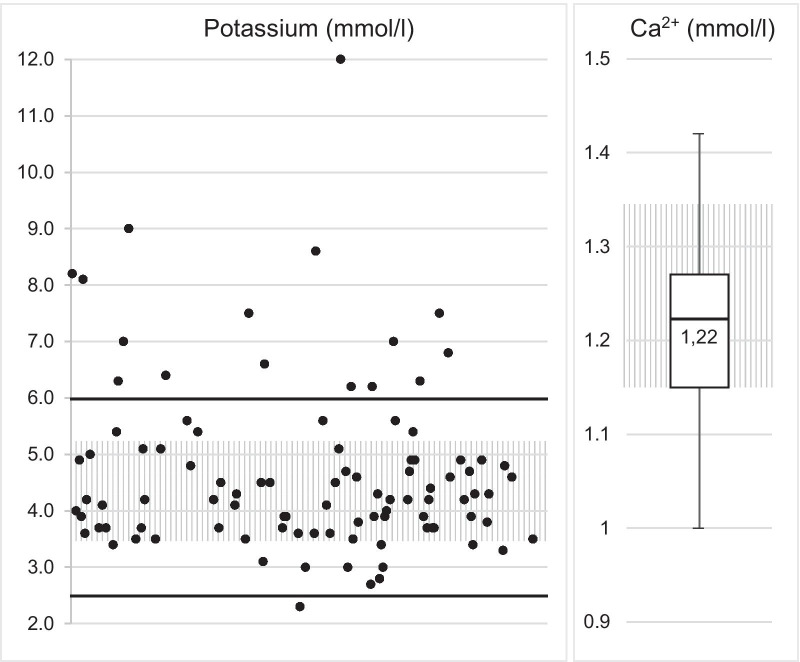
Electrolyte levels in POCT results of OHCA patients (normal range hatched, lines in bold: threshold for treatment according to ERC guideline 2015; upper/lower whiskers: maximum/minimum value; box: 75% to 25% quartile; median as line in the box)

The only significant difference found between venous and arterial samples was found in terms of carbon dioxide partial pressure (Fig. 1). A severe potassium imbalance (< 2.5 or > 6.0 mmol/l) was found in n = 17 (17%) measurements (Fig. 2). We found a significant correlation between hypokalaemia and an initial shockable rhythm on electrocardiogram (ECG) (shockable + hypokalaemia n = 9 (24%) vs non-shockable + hypokalaemia n = 2 (3%), *p* = 0.003). Patients with an initial non-shockable rhythm on ECG were more likely to be hyperkalaemic (non-shockable + hyperkalaemia n = 15 (25%) vs shockable + hyperkalaemia n = 2 (5%), *p* = 0.013).

**Fig. 3:**
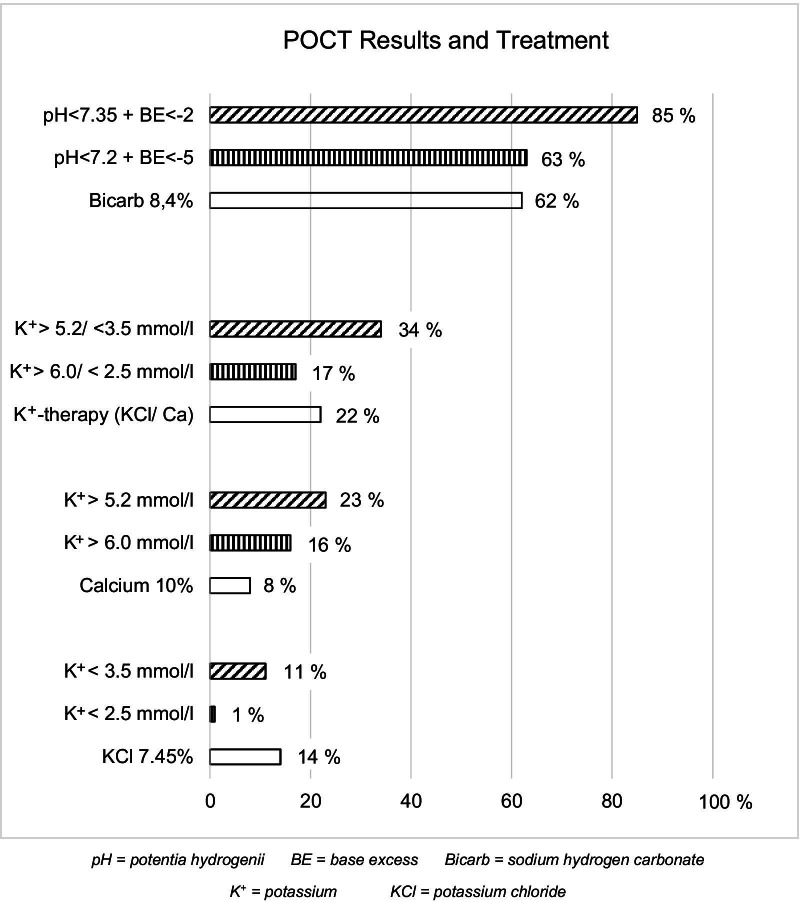
Abnormal POCT results and treatment of OHCA patients (n = 98)

Sodium bicarbonate 8.4% (“bicarb”) was administered to n = 61 (62%) of the n = 98 patients who underwent POCT and to n = 23 (14%) of the n = 165 who did not. Of the patients who underwent POCT, n = 22 (22%) received potassium-specific treatment. Potassium chloride was used in n = 14 (64%) of these cases. Of these, three patients with normal potassium levels (3.5–5.2 mmol/l) were treated with both potassium chloride and bicarb. In n = 8 (36%) cases, calcium chloride 10% was used; however, n = 11 (69%) patients with a measured potassium concentration of over 6.0 mmol/l were not treated in accordance with the guidelines by the emergency physician (Fig. 3). No other specific therapeutic measures aimed at reducing serum potassium were used.

**Fig. 4:**
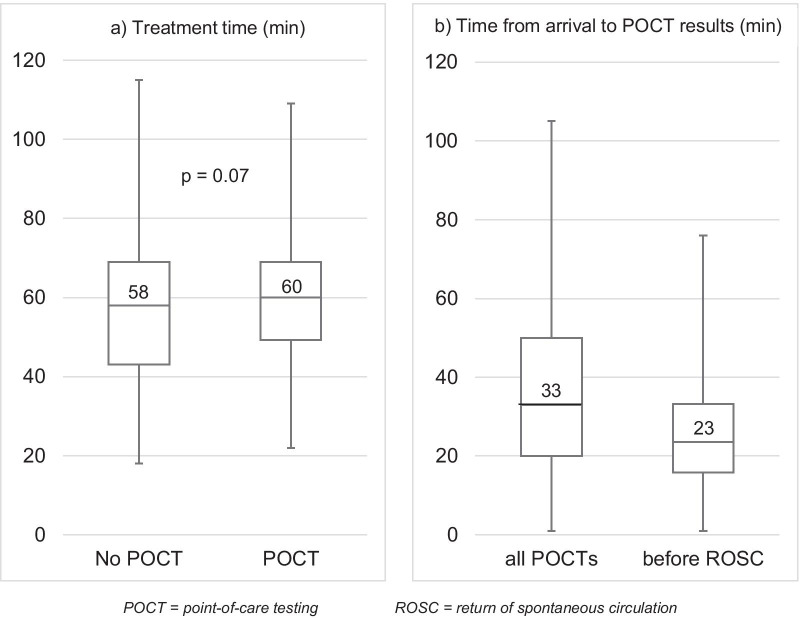
**a** Time from arrival on scene to start of transport by ambulance and **b** time from arrival on scene to availability of POCT results during the treatment of OHCA patients (upper/lower whiskers: maximum/minimum value; box: 75% to 25% quartile; median as line in the box)

The average time from arrival on scene to the result of the POCT becoming available during resuscitation and before the first ROSC was 26 min (± 15 min). The total pre-hospital treatment time from arrival at the scene of the emergency to the beginning of transport to the hospital by ambulance was 57 min on average (± 19 min) without POCT and 60 min (± 17 min) with POCT (*p* = 0.07) (Fig. 4).

**Fig. 5:**
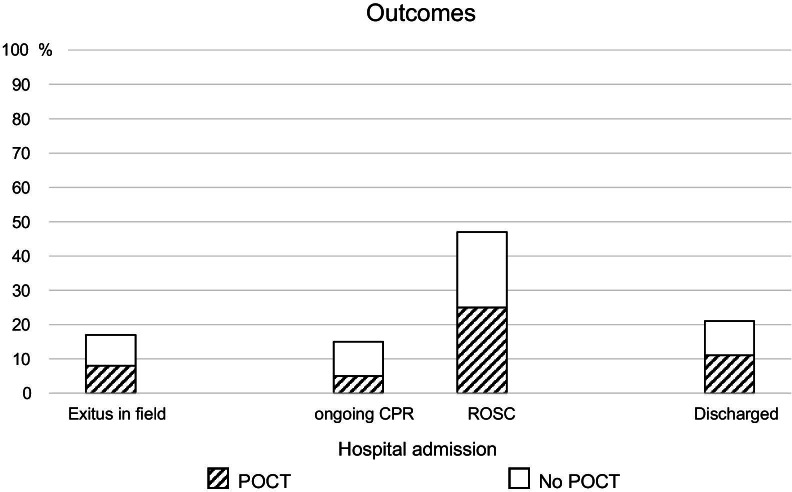
Percentages of different outcomes and ratios of POCT versus no POCT in relation to these outcomes (n = 263)

**Fig. 6:**
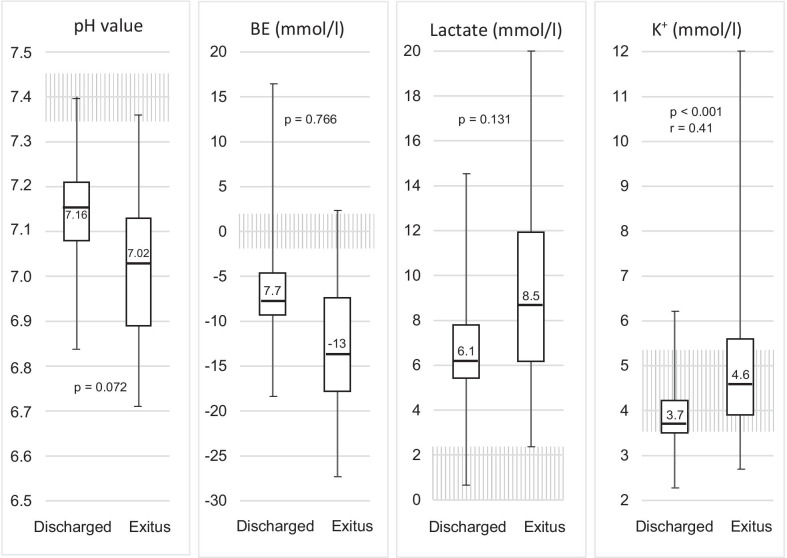
POCT laboratory results and outcome of OHCA patients (normal range hatched)

Out of the n = 263 OHCA patients included in this study, n = 55 (21%) were discharged from hospital. Of the n = 98 patients who underwent POCT, n = 29 (30%) survived to be discharged, compared with n = 26 (16%) of the n = 165 who did not undergo POCT (*p* = 0.01, Φ = 0.16) (Table 1, Fig. 5). The patients who did not survive tended to have had worse POCT results, although the difference was statistically significant only for potassium, with a moderate effect size (Fig. 6).

## Discussion

In this retrospective observational study of point-of-care testing in OHCA patients, we found frequent abnormalities in acid–base balance and electrolyte concentrations. Therapeutic measures were regularly carried as a result yet on-site treatment time was not extended significantly. An improved survival probability in OHCA patients who undergo POCT was observed.

### Data acquisition

Since all of the emergency medical service units (with emergency physicians) in this study region were operated by the same organisation and all of the vehicles were equipped with identical POCT equipment, there was no bias due to lack of equipment availability nor were there differences in measurements due to differences in training or differences between devices. Furthermore, since all patients in the study region are transported to the only available cardiac arrest centre, every patient in this study received similar hospital treatment and we were able to collect a complete set of data covering the entire course of treatment.

### Interpretation of the results

Abnormal pre-hospital POCT results can determine the choice of treatment. Given that such abnormal results occurred so frequently in this study, it seems important and useful to determine them as early as possible. In some cases, however, it must also be determined whether such values really do indicate subsequent treatment. In general, the more unfavourable a patient’s prognosis (due to a long period without resuscitation measures, for example), the further outside the reference range that patient’s POCT results are likely to be. In such cases, these early test results could be seen as an indicator of the prognosis rather than as a trigger for therapeutic measures.

The available evidence for administering buffer substances to resuscitation patients, for instance, is inconsistent. There are also no exact pH value limits to trigger buffering and the subject is controversial [13–18]. On the one hand, with increasing acidosis, the oxygen-haemoglobin dissociation curve shifts favourably within a certain range and oxygen release to peripheral tissues increases. On the other hand, acidosis reduces myocardial contractility, the threshold for possible ventricular fibrillation and the effectiveness of catecholamines [17, 19]. What is clear, however, is that alkalosis is harmful to resuscitation patients [20, 21]. What is more, buffering has the side effect of decreasing serum potassium concentration [22].

In this study, many patients had metabolic acidosis that required treatment. Patients who had collapsed unobserved and had an initial non-shockable rhythm on ECG were also often found to have pH and potassium levels indicative of acidosis and hyperkalaemia, respectively. These observations could potentially be interpreted as a basis for further treatment options if no POCT analyser is available. Taking such treatment measures without previously recorded baseline values could result in incorrect treatment, however.

There was no significant difference between venous and arterial samples in terms of the parameters that are relevant for buffering (pH, BE). Venous sampling thus seems sufficient.

While the need for buffering is controversially discussed, electrolyte imbalances should always be treated and there are clear relevant recommendations [10]. The frequency of hyperkalaemia found in this study as well as the deficit in terms of its treatment even when diagnosed early both suggest a lack of awareness of the issue, at least in the cases included in our study. The precise reasons for the deficit in treatment could not be determined in this study due to its design without questionnaires.

However, irrespective of these findings, for any measure taken in an emergency situation, the time and effort required must be weighed against the benefit. We found that treatment at the scene of the emergency was not delayed to any relevant extent by performing POCT. This was likely due to the common practice of taking a blood sample when placing an intravenous catheter, which does not require additional training on the part of the emergency medical team members and can thus easily be delegated. Venous sampling also happens to be more suitable for determining acid–base status [23, 24]. Arterial samples are needed for accurate assessments of gas exchange. Venous samples taken after the blood has passed through the capillary beds, on the other hand, provide more precise information about the acid–base status in the peripheral tissue.

The results of our study suggest that patients who underwent POCT had better overall outcomes. Patients who underwent POCT and survived to discharge also tended to have had better laboratory results on scene.

### Limitations

The retrospective design of this study without patient randomisation leaves some uncertainties when it comes to interpreting the results. Because the emergency medical service teams were not interviewed, their reasons for performing or, as the case may be, not performing POCT remain unclear. We compared the patient groups with and without pre-hospital point-of-care testing for a first overview of the topic even though these patient groups arose only by chance. Our assessment of the differences between these two groups is limited by these factors and any interpretations of our results must be cautious.

Because resuscitation patients are generally older and have acute pathologies, their prognosis is generally poor. Treatment for OHCA (including resuscitation) is a complex, multimodal process that is performed in a wide range of settings with many different influencing variables. All of these factors make it difficult to assess the effect of any individual measure, which, combined with the lack of proper patient randomisation in our study, means that our observations and evaluations must be interpreted with caution. However, we believe this makes it all the more important to optimise the treatment process wherever possible and appropriate.

### Perspectives

Whether POCT is globally applicable is open to debate. Germany has a comprehensive emergency medical service system with emergency physicians. We can therefore assume that emergency medical service teams in Germany have the specialist skills required to interpret the results of point-of-care testing and incorporate them into their response. Indeed, this additional measure appears to be primarily carried out by emergency medical service teams with advanced skills and training, which in itself presumably has a favourable effect on the patient’s overall prognosis. In general, we recommend using POCT at least in cases of suspected metabolic or electrolyte imbalances in the peri-arrest period. Further research should determine whether POCT can be used as part of the treatment process without negative consequences (or potentially even to the benefit of the patient) if the necessary training for emergency medical service teams is provided and standard procedures for its use as a diagnostic tool and the consequent complex treatment are established.

## Conclusions

This study shows that emergency physicians in the study region regularly perform POCT on resuscitation patients in the field. Such testing frequently revealed severe acidosis with metabolic imbalance as a contributory cause as well as abnormal potassium levels. Such findings are relevant for treatment and the fact that they occur so frequently demonstrates the relevance of this diagnostic tool in the pre-hospital acute setting. Since the overall duration of treatment was not adversely affected by pre-hospital point-of-care testing as an additional measure and because there was evidence of improved survival rates as a result, we believe that the benefits of this approach outweigh its disadvantages. We therefore conclude that the use of a POCT analyser could further optimise the treatment process for OHCA patients.

Further prospective, controlled and randomised trials on this topic are needed to verify our results.

## Data Availability

All data are available at the emergency centre of the University of Marburg.

## Abbreviations

Asys: Asystole
BE: Base excess
Bicarb: Sodium hydrogen carbonate
Ca: Calcium
CPR: Cardiopulmonary Resuscitation
ECG: Electrocardiogram
K^+^: Potassium
KCl: Potassium chloride
pCO_2_: Partial pressure of carbon dioxide
PEA: Pulseless electrical activity
PES: Pre-emergency status
pH: Potentia hydrogenii
POCT: Point-of-care testing
pVT: Pulseless ventricular tachycardia
ROSC: Return of spontaneous circulation
VF: Ventricular fibrillation
